# Anesthetic Management of Elderly Patients Undergoing Total Hip Arthroplasty for Femoral Neck Fractures: A Retrospective Observational Study

**DOI:** 10.7759/cureus.92898

**Published:** 2025-09-22

**Authors:** Mourad Ababou, Abderrahmane Elwali, Ayoub Bouaiyda, Achraf Jeddab, Hicham Hammadi, Mustapha Bensghir, Kamal Elmokhtari, Hicham Balkhi

**Affiliations:** 1 Department of Anesthesiology and Intensive Care, Military Hospital Mohammed V, Faculty of Medicine and Pharmacy of Rabat, Mohammed V University, Rabat, MAR; 2 Cardiothoracic Intensive Care Unit, Marie-Lannelongue Hospital, Paris, FRA; 3 Department of Orthopedics and Traumatology, Military Hospital Mohammed V, Faculty of Medicine and Pharmacy of Rabat, Mohammed V University, Rabat, MAR

**Keywords:** anesthesia outcomes, anesthetic techniques, elderly patients, postoperative complications, total hip arthroplasty

## Abstract

Introduction

Total hip arthroplasty (THA) for femoral neck fractures in elderly patients presents challenges for both surgeons and anesthesiologists. This study aimed to describe anesthesia-related issues and analyze risk factors for morbidity and mortality.

Materials and methods

This retrospective study was conducted over a four-year period in the Department of Anesthesiology, Mohammed V Military Teaching Hospital in Rabat, Morocco. It included patients aged ≥50 years who underwent surgery for femoral neck fractures and received THA.

Results

Seventy-nine patients were included, with a mean age of 66.8 ± 10.1 years; 81% were between 50 and 75 years old. Domestic accidents accounted for 62% of fractures. General anesthesia (GA) was administered to 55 patients (69.6%), while 30.4% received spinal anesthesia. Postoperative complications were primarily pain, anemia, and cognitive disorders. Mortality rates at 30 days, three months, and one year were 1.2% (1/79), 6.3% (5/79), and 15.2% (12/79), respectively. Increased mortality risk was associated with type 1 diabetes, anticoagulant use, intraoperative hypotension, perioperative blood transfusion, and longer hospitalization. Spinal anesthesia was associated with a reduced risk of intraoperative hypotension (p = 0.017) but a higher incidence of chronic pain compared with GA (33.3% vs. 12.7%, p = 0.032).

Conclusions

Outcomes in elderly patients undergoing THA for femoral neck fractures are influenced by comorbidities, prior treatments, and the perioperative course. The choice of anesthetic technique and timing of surgery appear to have a limited impact on prognosis.

## Introduction

Total hip arthroplasty (THA) is among the five most commonly performed surgical procedures worldwide. It is a functional prosthetic intervention involving the replacement of a damaged joint to improve patients’ quality of life [1]. THA is primarily indicated in elderly patients, largely due to age-related bone fragility. With the continuous aging of the global population, the frequency of this procedure has increased significantly, making it an important public health concern due to its notable morbidity and mortality rates [2].

This surgery can be performed under either general anesthesia (GA) or regional anesthesia (most commonly spinal anesthesia), with both techniques demonstrating overall comparable outcomes. However, optimal perioperative management is essential and should be undertaken urgently within a multidisciplinary framework. Key factors influencing morbidity and mortality include preoperative assessment, anesthetic technique selection, intraoperative management, and postoperative care. These considerations are particularly critical in elderly patients, who often present with multiple comorbidities and elevated perioperative risk.

The objective of this study is to evaluate the anesthetic management of elderly patients undergoing THA for femoral neck fractures at the Mohammed V Military Teaching Hospital in Rabat, Morocco.

## Materials and methods

This retrospective descriptive study included patients over 50 years of age who sustained a femoral neck fracture and underwent THA. Data were collected over a four-year period, from January 2020 to January 2024, at the Mohammed V Military Teaching Hospital in Rabat, Morocco. The study population consisted of patients admitted through the emergency department and operated on by one of the two orthopedic and trauma surgery departments at the institution.

Inclusion criteria were as follows: patients aged over 50 years who underwent surgery for femoral neck fracture during the study period and had complete, usable medical records. Exclusion criteria included patients under 50 years of age, femoral neck fractures occurring in the context of polytrauma, and cases with incomplete clinical documentation, particularly those lacking anesthetic protocols or operative reports.

Data were collected using a standardized extraction form (Appendix A) based on patient charts, surgical department registries, the DxCare electronic medical record system, pre-anesthesia consultation notes, intraoperative anesthetic monitoring sheets, and telephone follow-up interviews. Phone calls were conducted in June 2024 to supplement missing information and obtain follow-up data. A total of 94.4% of patients or their families responded. Follow-up focused on postoperative clinical course and the occurrence of complications, including cognitive disturbances, thromboembolic events, and infections.

All collected data were entered into Microsoft Excel (Microsoft Corporation, Redmond, WA, USA) and analyzed using JAMOVI software. Categorical variables were expressed as frequencies and percentages, while continuous variables with normal distribution were expressed as means with SDs. Univariate analysis was performed to assess associations between morbidity and mortality and both patient-related factors (e.g., advanced age and comorbidities) and management-related factors (e.g., surgical delay, anesthetic technique, and intraoperative events). Variables with statistically significant associations in univariate analysis were included in a multivariate model. Statistical tests used included the chi-square test or Fisher’s exact test for categorical variables and the Student’s t-test for continuous variables. A p-value < 0.05 was considered statistically significant.

## Results

A total of 218 patients underwent surgery for proximal femur fractures during the study period, representing 4% of the orthopedic-traumatology surgical activity. Of these, 125 medical records met the eligibility criteria (age ≥ 50 years and complete data) and were included in the analysis. Seventy-nine of these patients were treated with THA for femoral neck fractures.

The mean age of the patients was 66.8 ± 10.1 years (range: 50-93 years). Most patients (81%) were between 50 and 75 years of age. There was a slight male predominance, with a male-to-female ratio of 1.46 (59.5% male vs. 40.5% female).

Domestic accidents, including same-level falls and staircase accidents, were the most common mechanism of injury (62%). Road traffic accidents accounted for 6.3%, while prosthetic revision surgeries represented 24.1%. Other causes (e.g., work-related or sports injuries) made up 7.6%. Regarding the timing of surgical intervention, 31.6% of patients were operated on within 48 hours of trauma, 30.4% within one week, and 38% after more than one week.

Medical comorbidities were present in 60.8% of patients. The most frequent were hypertension (26.6%), diabetes mellitus (26.6%), ischemic heart disease (12.7%), and cognitive impairment (6.3%). Anesthetic history was positive in 57.7% of patients, most commonly with prior GA (41.7%) (Table 1).

**Table 1:** Patients’ medical history

Medical comorbidities	Condition	Frequency (n, %)
Metabolic history	Type I diabetes	21 (26.5%)
	Type II diabetes	5 (6.3%)
	Dyslipidemia	5 (6.3%)
	Thyroid disorders	1 (1.2%)
Respiratory history	Asthma	13 (16.4%)
	Chronic obstructive pulmonary disease	3 (3.8%)
	Tuberculosis	2 (2.5%)
	Pulmonary embolism	1 (1.2%)
	COVID-19 infection	2 (2.5%)
Cardiovascular history	Hypertension	21 (26.5%)
	Ischemic heart disease	5 (6.3%)
	Heart failure	5 (6.3%)
	Pacemaker implantation	1 (1.2%)
Neurological history	Cognitive impairment	5 (6.3%)
	Stroke	1 (1.2%)
	Parkinson’s disease	1 (1.2%)
	Depression	1 (1.2%)
Oncological history	History of malignancy	4 (5.1%)
Renal and urological history	Chronic kidney disease	2 (2.5%)
	Benign prostatic hyperplasia	2 (2.5%)

Pre-anesthetic evaluation showed that 49.4% of patients were classified as ASA I, 36.7% as ASA II, and 13.9% as ASA III. Functional capacity (MET > 4) was preserved in 88.6% of patients, while 11.4% had limited functional status. Predictors of difficult intubation were present in 20.3%. Baseline laboratory and imaging workups were performed for all patients. ECG abnormalities were observed in 14.7%, and transthoracic echocardiography was conducted in 18.7%, with left ventricular dysfunction identified in three cases. Laboratory findings included anemia in 20.2%, coagulation disorders in 7.5%, and renal impairment in 8.8%.

Pain management was primarily pharmacological, consisting of paracetamol, nonsteroidal anti-inflammatory drugs (NSAIDs), and nefopam. Femoral nerve blocks were used in three cases, and iliac fascia blocks in two. Only 12.6% of patients received premedication, equally distributed between benzodiazepines and hydroxyzine. Thromboprophylaxis with low-molecular-weight heparin (LMWH) was administered in 93.6% of patients; five patients were already receiving anticoagulation therapy upon admission.

Standard intraoperative monitoring was applied to all patients, with invasive arterial monitoring in 2.53% of cases. Capnography was used in all patients undergoing GA (69.6%). All patients received prophylactic antibiotics 30 minutes prior to incision. Cefazolin was the most frequently used (76.8%), followed by amoxicillin-clavulanate (21.8%). Vancomycin and ciprofloxacin were each administered to one patient (0.7%). GA was used in 69.6% of cases, and spinal anesthesia in 30.4%. Induction for GA typically included propofol (93.6%), rocuronium, and fentanyl; ketamine was used in 5.4%. Maintenance was primarily with volatile anesthetics, mainly isoflurane (84.6%) and, to a lesser extent, sevoflurane (15.4%). Total intravenous anesthesia with propofol was used in three patients. Intraoperative hypotension occurred in 49.1% of patients under GA and in 16.7% of those under spinal anesthesia.

The mean operative time was 121 ± 61.3 minutes overall, slightly longer in patients receiving GA (125 ± 67.6 minutes) compared with spinal anesthesia (113 ± 42.4 minutes). Hospital stays ranged from three to 35 days, with a median of seven days and a mean of 9.56 days.

Postoperative complications included chronic pain (19%), anemia (15.2%), postoperative cognitive dysfunction (POCD; 15.2%), infection (3.8%), and respiratory complications (2.5%). One thromboembolic event and one case of postoperative COVID-19 were reported. Surgical revision was required in 6.31% of cases. In-hospital mortality was 1.2%. Mortality rates at 30 days, three months, and one year were 1.2%, 6.3%, and 15.2%, respectively.

At three months postoperatively, univariate analysis revealed significant associations between mortality and intraoperative blood transfusion (p = 0.028, OR = 8.45 (1.26-56.56)) and length of hospital stay (p = 0.024, OR = 1.113 (1.01-1.22)). In multivariate analysis, adjusting for age, cardiac comorbidities, hypotension, transfusion, and length of stay, only hospital stay remained significantly associated with three-month mortality (p = 0.037, OR = 1.13 (1.00-1.26)). Table 2 summarizes the results of the univariate and multivariate analyses.

**Table 2:** Factors associated with three-month mortality The threshold for statistical significance was set at p < 0.05. GA, general anesthesia; MET, metabolic equivalent of task; VTE, venous thromboembolism

Variable	Univariate analysis			Multivariate analysis		
	OR	95% CI	p-Value	OR	95% CI	p-Value
Demographic factors						
Sex: M-F	0.425	0.06-2.63	0.351	-	-	-
Age	1.037	0.95-1.13	0.396	1.067	0.95-1.19	0.227
Preoperative factors						
Hypertension	0.662	0.07-6.29	0.72	-	-	-
Diabetes T1 - non	3.31 × 10⁻⁸	-	0.996	-	-	-
Diabetes T2 - non	3.31 × 10⁻⁸	-	0.995	-	-	-
Cardiopathy	1.777	0.18-17.72	0.624	0.744	0.05-10.77	0.828
VTE	3.29 × 10⁻⁷	-	0.995	-	-	-
Preop cognitive disorders	1.18 × 10⁻⁷	-	0.996	-	-	-
Anticoagulants	4.312	0.38-48.11	0.235	-	-	-
Antiplatelets	1.14 × 10⁻⁷	-	0.995	-	-	-
Management >48 hours	1.449	0.22-9.27	0.695	-	-	-
Preop anemia	4.31 × 10⁻¹⁰	-	0.998	-	-	-
Renal failure	1.14 × 10⁻⁷	-	0.995	-	-	-
MET<4	1.11 × 10⁻⁷	-	0.994	-	-	-
Per- and postoperative factors						
Hypotension	6.37	0.67-60.04	0.106	5.03	0.37-67.56	0.222
Blood transfusion	8.454	1.26-56.56	0.028	5.43	0.59-49.27	0.131
Post-op cognitive disorders	4.2	0.62-28.34	0.141	-	-	-
GA	0.605	0.09-3.89	0.597	-	-	-
Spinal anesthesia	1.65	1.26-10.60	0.597	-	-	-
Surgical duration	1.004	0.99-1.02	0.523	-	-	-
Hospital stay duration	1.113	1.01-1.22	0.024	1.128	1.00-1.26	0.037

For one-year mortality, univariate analysis identified type 1 diabetes (p = 0.022), anticoagulant use (p = 0.015), intraoperative hypotension (p = 0.029), and length of hospital stay (p = 0.055) as significant factors. In multivariate analysis, type 1 diabetes (p = 0.004) and intraoperative hypotension (p = 0.022) were independently associated with one-year mortality. Table 3 summarizes the results of the univariate and multivariate analyses.

**Table 3:** Factors associated with one-year mortality The threshold for statistical significance was set at p < 0.05. GA, general anesthesia; MET, metabolic equivalent of task; VTE, venous thromboembolism

Variables	Univariate analysis			Multivariate analysis		
	OR	95% CI	p-Value	OR	95% CI	p-Value
Demographic factors						
Sex: M-F	0.945	0.27-3.29	0.929	0.992	0.81-1.10	0.87
Age	0.984	0.92-1.05	0.602	-	-	-
Preoperative factors						
Hypertension	0.907	0.22-3.73	0.893	-	-	-
Diabetes						
T1 - no	6.25	1.29-30.00	0.022	193.63	5.38-∞	0.004
T2 - no	0.993	-	0.933	2.22E-08	6967.12	0.997
Heart disease	1.475	0.27-7.98	0.652	-	-	-
VTE	3.40E-07	-	0.991	1.22E-10	-	0.998
Pre-op cognitive disorders	4.267	0.68-28.79	0.136	-	-	-
Anticoagulants	10.83	1.58-73.91	0.015	9.44E+07	-	0.997
Antiplatelets	2.48	0.42-14.57	0.315	-	-	-
Management >48 hours	0.38	0.07-1.89	0.239	-	-	-
Pre-op anemia	2.31E+07	-	0.997	-	-	-
Renal failure	2.48	0.42-14.60	0.239	-	-	-
MET<4	3.39	0.71-16.01	0.123	-	-	-
Per- and postoperative factors						
Hypotension	4.87	1.17-20.16	0.029	19.93	1.54-257	0.022
Blood transfusion	0.917	0.17-4.73	0.917	-	-	-
Post-op cognitive disorders	6.122	1.52-24.56	0.011	6.93	0.35-137	0.203
GA	1.37	0.33-5.58	0.661	-	-	-
Spinal anesthesia	0.73	1.79-2.97	0.661	-	-	-
Surgery duration	1	0.99-1.013	0.515	-	-	-
Hospital stay duration	1.073	0.99-1.15	0.055	0.987	0.853-1.14	0.866

Comparison of morbidity and mortality between spinal anesthesia and GA showed a significantly higher incidence of intraoperative hypotension in the GA group (49.1%) compared with the spinal anesthesia group (19%) (p = 0.017; OR = 4.10; 95% CI: 1.22-13.8). No statistically significant difference was observed between the two groups regarding postoperative anemia (12.5% in spinal anesthesia vs. 16.4% in GA; p = 0.66) or cognitive impairment (12.5% vs. 20%; p = 0.071). However, chronic pain was significantly more frequent in the spinal anesthesia group (33.3%) than in the GA group (12.7%) (p = 0.032; OR = 0.292; 95% CI: 0.091-0.93). No significant differences were found in postoperative infection rates (4.2% vs. 1.8%; p = 0.518) or in mortality at three months (8.7% vs. 5.5%; p = 0.628), one year (12.5% vs. 16.4%; p = 0.747), or three years (20.8% vs. 25.5%; p = 0.659). Table 4 summarizes the results of the statistical analysis comparing spinal and GA.

**Table 4:** Results of statistical analysis comparing morbidity and mortality between spinal anesthesia and GA The threshold for statistical significance was set at p < 0.05. GA, general anesthesia

Variables	Spinal anesthesia	GA	p-Value and OR (95% CI)
Hypotension	19%	49.1%	p = 0.017, OR = 4.10 (1.22-13.8)
Postoperative anemia	12.5%	16.4%	p = 0.66, OR = 1.37 (0.336-5.58)
Cognitive disorders	4.2%	20%	p = 0.071, OR = 5.75 (0.638-47.3)
Chronic pain	33.3%	12.7%	p = 0.032, OR = 0.292 (0.091-0.93)
Postoperative infections	4.2%	1.8%	p = 0.518, OR = 0.426 (0.02-7.11)
Death at three months	8.7%	5.5%	p = 0.628, OR = 1.65 (0.257-10.6)
Death at one year	12.5%	16.4%	p = 0.747, OR = 0.730 (0.179-2.98)
Death at three years	20.8%	25.5%	p = 0.659, OR = 1.35 (0.408-4.13)

Similarly, when comparing early (<48 hours) versus delayed (>48 hours) management, the incidence of intraoperative hypotension was higher in the delayed group (45.1%) compared with the early group (32.2%), although this difference was not statistically significant (p = 0.275; OR = 0.573; 95% CI: 0.21-1.57). No significant differences were observed between the two groups regarding postoperative anemia (8% vs. 18.5%; p = 0.226), chronic pain (16% vs. 20.4%; p = 0.764), or postoperative infection (0% vs. 3.7%; p = 0.33). Cognitive disorders were more frequent in the delayed group (20.4%) compared with the early group (4%), but this difference did not reach statistical significance (p = 0.091; OR = 0.163; 95% CI: 0.019-1.34). Likewise, no significant differences were observed in mortality at three months (8% vs. 5.7%; p = 0.653), one year (8% vs. 18.5%; p = 0.226), or three years (20% vs. 25.9%; p = 0.56) between the early and delayed management groups. Table 5 summarizes the results of this comparison.

**Table 5:** Statistical analysis comparing morbidity and mortality between early management (<48 hours) and delayed management (>48 hours) The threshold for statistical significance was set at p < 0.05.

Outcome	<48 hours (%)	>48 hours (%)	p-Value	OR (95% CI)
Hypotension	32	45.1	0.275	0.573 (0.210-1.57)
Postoperative anemia	8	18.5	0.226	0.383 (0.077-1.89)
Cognitive disorders	4	20.4	0.091	0.163 (0.019-1.34)
Chronic pain	16	20.4	0.764	0.745 (0.212-2.62)
Postoperative infection	0	3.7	0.33	0.412 (0.01-8.9)
Three-month mortality	8	5.7	0.653	0.690 (0.10-4.42)
One-year mortality	8	18.5	0.226	2.61 (0.52-12.9)
Three-year mortality	20	25.9	0.56	0.714 (0.225-2.26)

## Discussion

Preoperative assessment for hip surgery considers the procedure to be of intermediate risk. Current European guidelines recommend measuring troponin in patients with cardiovascular disease or risk factors (including age ≥65) before intermediate- or high-risk surgery, and again 24-48 hours postoperatively, to enable early detection of perioperative myocardial infarction. Echocardiography before intermediate-risk surgery is indicated only in cases of reduced functional capacity, abnormal ECG, elevated NT-proBNP/BNP, or at least one clinical risk factor.

Early surgical intervention improves outcomes by reducing hospital stay, complications, and mortality, while facilitating return to independence [3]. Studies variably define early surgery as within 24-48 hours of admission.

Pain management should be precise, using appropriate scales for older adults such as the Visual Analogue Scale [4]. Multimodal analgesia, which combines peripheral and central mechanisms, is preferred. Paracetamol is first-line in the elderly; NSAIDs may be used cautiously, and opioids only when other methods are insufficient [5]. Peripheral nerve blocks, such as femoral or iliofascial blocks, ideally ultrasound-guided, reduce postoperative pain, decrease opioid requirements, and improve early mobilization. Intra-articular or subcutaneous infiltrations are not recommended due to insufficient evidence [6].

Preoperative medication management requires careful optimization without delaying surgery. In our study, 25.3% of patients received antihypertensives, 16.5% oral antidiabetics, 15.2% insulin, and 8.9% antiplatelet agents. Large cohort studies show that polypharmacy is common in elderly patients [7]. Beta-blockers and statins should generally be continued, while ACE inhibitors and ARBs may be withheld 12 hours preoperatively unless prescribed for heart failure.

Anticoagulant management should follow current guidelines. DOACs should be stopped two to three days before surgery if renal function is normal, with LMWH bridging in patients at high thromboembolic risk. Therapeutic LMWH should be stopped 24 hours preoperatively, while VKAs should be discontinued five days prior and bridged with LMWH [8]. Antiplatelet therapy should be individualized: aspirin may be continued in patients with high thrombotic risk, whereas clopidogrel is generally stopped five to seven days before surgery.

Elderly patients with femoral neck fractures are at high thromboembolic risk, particularly those over 75. Additional risk factors include prior thromboembolism, surgery lasting more than two hours, varicose veins, post-thrombotic syndrome, or delayed surgery beyond 48 hours [9]. In our cohort, all patients received perioperative anticoagulation, with only 1.3% developing deep vein thrombosis, consistent with published rates. Thromboprophylaxis should begin preoperatively if surgery is delayed for more than 12 hours and continue postoperatively for at least 10-14 days, and up to 35 days in high-risk patients, preferably with prophylactic-dose LMWH. Alternatives include fondaparinux, low-dose unfractionated heparin, or aspirin, while intermittent pneumatic compression may be used when anticoagulation is contraindicated.

THA is classified as Altemeier Class I surgery. The baseline risk of surgical site infection ranges from 3% to 5% but decreases to 0.4-1% with appropriate antibiotic prophylaxis. Recommended agents include cefazolin, clindamycin, or vancomycin, administered 30-60 minutes before incision and repeated every four to six hours depending on drug half-life, with discontinuation after 24 hours except in specific cases [10]. Individualized prophylaxis is recommended for patients with prior joint or skin infections, inflammatory bowel disease, or immunosuppression.

THA is generally performed in the lateral decubitus position, with a mean operative time of 80 minutes. Orotracheal intubation is required for airway protection and is often facilitated by neuromuscular blockade in elderly or frail patients. In our study, GA was associated with higher risks of perioperative hypotension (p = 0.017), chronic pain (p = 0.032), and cognitive impairment (p = 0.071). Similar findings were reported by Anwer et al. [11] and in a prospective Asian cohort [12], which showed increased risks of stroke (OR = 1.18, 95% CI: 1.07-1.31; p = 0.001), respiratory failure (OR = 2.71, 95% CI: 2.38-3.01; p < 0.001), and ICU admission. The REGAIN trial [13] demonstrated that spinal anesthesia reduced the incidence of renal failure and ICU admission compared with GA.

Spinal and peripheral nerve blocks are the main locoregional techniques. Peripheral blocks are rarely used intraoperatively but can provide effective postoperative analgesia. A prospective study conducted at Military Hospital Mohammed V [14] comparing single-shot versus continuous spinal anesthesia for THA found higher rates of hypotension and greater vasopressor requirements in the single-shot group (p = 0.025 and p < 0.001, respectively). Advanced age, chronic hypertension, and lower pre-induction systolic blood pressure were identified as risk factors for perioperative hypotension [15]. Shin et al. [16] reported that continuous spinal anesthesia provided a more stable hemodynamic profile than GA with sevoflurane in elderly patients. A meta-analysis by Chen et al. [17] found no significant differences in postoperative pneumonia, heart failure, myocardial infarction, acute kidney injury, stroke, delirium, or thromboembolic events between spinal and GA, although spinal anesthesia was associated with reduced rates of acute respiratory failure and hospital readmission. The choice of anesthetic technique should therefore be individualized based on patient history, anesthesiologist expertise, operative conditions, and patient preference.

Elderly patients are particularly susceptible to anesthetic-induced hypotension, which increases the risk of delirium, acute kidney injury, and cardiac arrhythmias. Blood loss during THA is estimated at approximately 30% of total blood volume, rising to 43% in revision procedures. In our study, 15.2% of patients were anemic at admission, and 17.7% required transfusion. Preexisting or trauma-related anemia affects 10-40% of patients with femoral neck fractures [18]. The FOCUS trial [19] found no benefit of liberal transfusion strategies (Hb < 10 g/dL) over restrictive strategies (Hb < 8 g/dL), with meta-analyses supporting increased postoperative complications in patients managed with liberal transfusion thresholds. Intravenous iron supplementation combined with a restrictive transfusion strategy is effective, while tranexamic acid reduces transfusion requirements without increasing thrombotic risk.

Cemented THA carries a risk of rare but severe complications, including cardiac arrest, hypoxemia, and circulatory collapse. Intraoperative transesophageal echocardiography has demonstrated that most emboli originate from the medullary cavity due to high intramedullary pressure rather than cement toxicity [20]. Clinical presentation typically begins with right heart failure, which may progress to global cardiac failure. Management is supportive, consisting of oxygenation, fluid resuscitation, and catecholamines. Preventive strategies include intramedullary drainage, cavity lavage, aspiration before cementing, optimal oxygenation and normovolemia, adequate analgesia, and the use of uncemented prostheses.

THA generally lasts about two hours, which increases the risk of peri-anesthetic hypothermia. The initial phase results from core-to-peripheral heat redistribution and can be reduced with preoperative skin warming or vasodilators such as calcium channel blockers [21]. During the second phase, forced-air warming blankets are effective, with their success influenced by both coverage area and duration of use. These measures reduce postoperative shivering, bleeding, and infection [22]. Patient positioning also affects cardiopulmonary function; lateral decubitus with the operated limb above the right atrium may increase the risk of gas embolism in patients with a patent foramen ovale. Additionally, lateral positioning alters ventilation-perfusion ratios, predisposing the dependent lung to atelectasis. Common positioning-related injuries include brachial plexus, sciatic nerve, and suprascapular nerve compression, as well as injury to structures within Scarpa’s triangle.

POCD includes two types of impairments: acute, transient delirium with fluctuating attention and orientation, and more subtle, long-term cognitive deficits in elderly patients. POCD is often incorrectly attributed solely to anesthesia, although multiple perioperative factors contribute. In our study, the incidence of POCD was 15.2%, with a higher, though not statistically significant, occurrence after GA (p = 0.071). POCD was significantly associated with prolonged hospitalization (p < 0.001) and chronic pain (p < 0.001). Risk factors include intraoperative hypotension and hypoxemia, both of which reduce cerebral perfusion. Two retrospective studies [23] found no significant difference in postoperative stroke incidence between general and regional anesthesia (OR = 1.08; 95% CI: 0.82-1.4).

Postoperative cardiovascular complications are influenced by advanced age, preexisting cardiovascular disease, comorbidities, renal impairment, intraoperative hypotension, prolonged surgery and anesthesia, and excessive use of vasopressors or inotropes [24]. A study in Marrakech identified age ≥85 years, a history of cardiac disease, and ASA class 3 as independent predictors of cardiovascular complications. Venous thromboembolism (VTE) is the most common cardiovascular complication after THA, with atrial fibrillation, congestive heart failure, and myocardial infarction also reported [25]. Additional risk factors include male sex, obesity, hypertension, diabetes, and prior cardiovascular disease.

Respiratory complications after THA occur more frequently in elderly patients, those with comorbidities, and patients receiving GA. Memtsoudis et al. reported an overall complication rate of 4.7% [25]. In our cohort, prolonged hospitalization and ICU admission were associated with higher rates of respiratory complications (p = 0.005 and p = 0.05), highlighting the importance of early mobilization.

Thromboembolic complications are more common in patients over 60 years old, with obesity, smoking, previous thrombosis, and longer surgery duration as significant risk factors. Parvizi et al. reported an overall VTE rate of 2.1% in patients over 75 years, higher in those with a history of thrombosis or prolonged surgery [26]. In our series, one patient (1.3%) developed deep vein thrombosis.

Mortality risk factors include high ASA score, prolonged hospitalization, severe osteoporosis, female sex, and advanced age, as well as BMI, race, smoking, dyspnea, and functional or neurological status [27]. Morbidity and perioperative mortality are more strongly influenced by baseline clinical status than by age alone. Regional (spinal) anesthesia may reduce in-hospital mortality compared to GA, although differences in 30-day mortality are not significant [28]. In our study, 30-day mortality was 1.2%, and three-month mortality was 6.3%. Independent predictors of mortality included patient comorbidities, delayed management, length of hospitalization, and the need for blood transfusion.

This study has several limitations. Its retrospective, single-center design and relatively small sample size may limit the generalizability of the findings and reduce the statistical power of subgroup analyses. In addition, incomplete data on long-term functional outcomes restricted the scope of the analysis, and residual confounding cannot be excluded despite multivariate adjustment.

## Conclusions

Femoral neck fractures represent a significant public health concern due to their severity and increasing incidence. Given the unique characteristics of elderly patients, several professional societies have developed recommendations for individualized management, aiming to improve outcomes. Implementing specific protocols is therefore essential to reduce morbidity and mortality. To optimize perioperative care, several key actions are highlighted in our study. First, reducing time to surgery is crucial, which requires raising awareness among patients and families to seek prompt medical attention after hip fractures while avoiding unnecessary investigations that may delay intervention. Effective management of chronic medications, such as anticoagulants, is also critical and should not postpone surgery. Appropriate use of antibiotic prophylaxis and thromboprophylaxis remains a cornerstone of management.

Establishing dedicated care pathways and reorganizing emergency operating room schedules may further enhance patient care. Regarding anesthetic technique, the approach should be tailored to each patient according to their individual risk profile. Greater use of regional anesthesia, both pre- and postoperatively, and broader dissemination of peripheral nerve block techniques, particularly the iliofascial block, among all practitioners involved in patient care are strongly encouraged.

## Appendices

Appendix A: Data collection sheet

I. Socioepidemiological data

Name: _________________________

First name: ____________________

Sex: ☐ M ☐ F

Age: _____

II. Medical history

Medical history: ☐ No ☐ Yes

If yes:

Hypertension

Diabetes

Heart disease

Thromboembolic disease

Cognitive disorders

Allergies

Others: ____________________

Current medication: ☐ No ☐ Yes

If yes: Medication (class): ____________________

Anesthesia history: ☐ No ☐ Yes

If yes:

☐ GA

☐ Spinal anesthesia

Incidents: ☐ No ☐ Yes

III. Clinical data

Cause of trauma:

☐ Road accident

☐ Domestic accident

☐ Revision

☐ Others: ____________________

Management:

☐ Emergency

☐ Elective

Admission delay:

☐ <12h

☐ 12h-24h

☐ 24h-48h

☐ 48h-5d

☐ >5d

Treatment delay:

☐ <24h

☐ 24h-48h

☐ 48h-5d

☐ >5d

IV. Pre-anesthetic assessment

ASA (1-5): _____

MET score: ☐ <4

Difficult intubation criteria: ☐ No ☐ Yes

If yes:

Mouth opening

TMD

Teeth

Spinal stiffness

Mallampati

Biological disorders:

☐ Anemia

☐ Coagulation disorder

☐ Renal failure

☐ Infectious syndrome

☐ Electrolyte disorder

☐ Others: ____________________

Investigations:

ECG: ☐ Normal ☐ Abnormal

Echocardiography: ☐ Normal ☐ Abnormal

Imaging:

Chest X-ray: ____________________

Pelvic X-ray: ____________________

CT scan: ____________________

MRI: ____________________

Others: ____________________

V. Patient preparation

Medication management: ____________________

Additional treatment: ____________________

Pain management:

Drug: ____________________

Nerve block: ____________________

Thromboprophylaxis: ____________________

Premedication: ____________________

VI. Anesthetic protocol

Volume expansion: ____________________

Monitoring: ____________________

Antibiotic prophylaxis:

☐ 1st Gen Ceph

☐ 3rd Gen Ceph

☐ Penicillin

☐ Others: ____________________

Anesthetic technique:

☐ GA

☐ Regional anesthesia

If GA:

Maintenance: ☐ TIVA ☐ Balanced anesthesia

If balanced anesthesia: ☐ Isoflurane ☐ Sevoflurane

If spinal anesthesia:

☐ Standard spinal

☐ Continuous spinal

Intraoperative events:

Hypotension: ☐ No ☐ Yes

Vasopressors: ☐ No ☐ Yes

Transfusion: ☐ No ☐ Yes

Tranexamic acid: ☐ No ☐ Yes

Total duration of surgery (min): __________

Complications: ☐ No ☐ Yes: ____________________

Recovery: ☐ Normal ☐ ICU

VII. Postoperative treatment

Analgesia:

Analgesics: ☐ Morphine ☐ NSAIDs ☐ RA-morphine ☐ Block

Antibiotics: ____________________

Thromboprophylaxis: ____________________

VIII. Outcome

Favorable hospital course: ☐ Yes ☐ No

If no:

Cognitive disorders

Thromboembolism

Anemia

Infections

Metabolic disorders

Hydro-electrolytic disorders

Postoperative respiratory complication (PRC)

Cardiac complications

Bedsores

Others: ____________________

Length of hospital stay: _____ days

Telephone contact reachable? ☐ Yes ☐ No

Reported problems:

Osteosynthesis material failure

Chronic pain

Bone infection

Reoperation on site

Other hospitalizations: ____________________

Normal walking: ____________________

Others: ____________________

Death? ☐ No ☐ Yes

In-hospital?

30 days?

3 months?

1 year?
